# Validity of simplified, calibration-less exercise intensity measurement using resting heart rate during sleep: a method-comparison study with respiratory gas analysis

**DOI:** 10.1186/s13102-019-0140-x

**Published:** 2019-11-04

**Authors:** Hirotaka Matsuura, Masahiko Mukaino, Yohei Otaka, Hitoshi Kagaya, Yasushi Aoshima, Takuya Suzuki, Ayaka Inukai, Emi Hattori, Takayuki Ogasawara, Eiichi Saitoh

**Affiliations:** 1 Department of Rehabilitation Medicine I, School of Medicine, Fujita Health University, Toyoake, Japan; 2 Department of Rehabilitation Medicine, Fujita Health University Hospital, Toyoake, Japan; 3 NTT Device Innovation Center, NTT Basic Research Laboratories, NTT Corporation, Atsugi, Japan

**Keywords:** Heart rate, Wearable devices, Percent oxygen consumption reserve, Exercise intensity, 6-min walk test

## Abstract

**Background:**

The recent development of wearable devices has enabled easy and continuous measurement of heart rate (HR). Exercise intensity can be calculated from HR with indices such as percent HR reserve (%HRR); however, this requires an accurate measurement of resting HR, which can be time-consuming. The use of HR during sleep may be a substitute that considers the calibration-less measurement of %HRR. This study examined the validity of %HRR on resting HR during sleep in comparison to percent oxygen consumption reserve (%VO_2_R) as a gold standard. Additionally, a 24/7%HRR measurement using this method is demonstrated.

**Methods:**

Twelve healthy adults aged 29 ± 5 years underwent treadmill testing using the Bruce protocol and a 6-min walk test (6MWT). The %VO_2_R during each test was calculated according to a standard protocol. The %HRR during each exercise test was calculated either from resting HR in a sitting position (%HRR_sitting_), when lying awake (%HRR_lying_), or during sleep (%HRR_sleeping_). Differences between %VO_2_R and %HRR values were examined using Bland-Altman plots. A 180-day, 24/7%HRR measurement with three healthy adults was also conducted. The %HRR values during working days and holidays were compared.

**Results:**

In the treadmill testing, the mean difference between %VO_2_R and %HRR_sleeping_ was 1.7% (95% confidence interval [CI], − 0.2 to 3.6%). The %HRR_sitting_ and %HRR_lying_ values were 10.8% (95% CI, 8.8 to 12.7%) and 7.7% (95% CI, 5.4 to 9.9%), respectively. In the 6MWT, mean differences between %VO_2_R and %HRR_sitting_, %HRR_lying_ and %HRR_sleeping_ were 12.7% (95% CI, 10.0 to 15.5%), 7.0% (95% CI, 4.0 to 10.0%) and − 2.9% (95% CI, − 5.0% to − 0.7%), respectively. The 180-day, 24/7%HRR measurement presented significant differences in %HRR patterns between working days and holidays in all three participants.

**Conclusions:**

The results suggest %HRR_sleeping_ is valid in comparison to %VO_2_R. The results may encourage a calibration-less, 24/7 measurement model of exercise intensity using wearable devices.

**Trial registration:**

UMIN000034967.

Registered 21 November 2018 (retrospectively registered).

## Background

The measurement of exercise intensity can be used to monitor energy consumption or appropriate workloads during exercise [1]. Oxygen consumption (VO_2_) and heart rate (HR) can be used to assess exercise intensity [2]. Using respiratory gas analyzers, exercise intensity can be assessed with indices such as percent VO_2_ reserve (%VO_2_R) or percent maximum VO_2_ [3]. These indices are usually evaluated during exercise testing (e.g., the Bruce protocol or the Balke protocol) and are used when prescribing exercises [4, 5]. Indices of exercise intensity during exercise testing provide accurate information on an individual’s cardiovascular and pulmonary capacity for a specific workload. However, such testing requires specialized settings. In addition, these tests are sometimes difficult to perform for individuals with disabilities, because limited mobility may mask cardiovascular and respiratory aspects of functional capacity.

HR is widely used to monitor exercise intensity [6, 7]. In particular, percent HR reserve (%HRR) is frequently used as an index of exercise intensity [8] and has been correlated and is in good agreement with %VO_2_R, although %HRR seems to be slightly lower in low exercise intensities [9, 10]. Both %VO_2_R and %HRR are suitable measures for exercise prescription [11, 12]. The merit of using HR lies in its ease of use; it can be monitored in various settings and is not limited to specialized settings. In addition to their HR during exercise, an individual’s resting and maximum HR are needed to calculate %HRR. The maximum HR should ideally be measured by exercise testing, but there is an easy alternative method to estimate maximum HR with a simple equation using an individual’s age [13]. On the other hand, the resting HR should actually be measured, and the accuracy of resting HR is important for precise calculation of the exercise intensity. HR can easily be influenced by mental state at the time of measurement and/or activities immediately before measurement. Therefore, sufficient pre-measurement rest and abstention from exercise is needed to obtain an accurate resting HR [14]. However, in usual clinical settings, it may not always be possible to provide sufficient time for a subject for resting HR measurement. One possible solution may be to measure the HR during sleep, which should be lower and more stable than that during the awake condition [15]. Recording HR during sleep may present less measurement errors related to psychological or physical factors, which are difficult to eliminate when the measurements are taken while the individual is awake. In addition, longer periods of HR measurement may improve the accuracy of resting HR measurements. Recent technological developments have enabled the easy monitoring of HR using wearable devices. For example, wrist-band type measurement devices or smart clothing systems have reportedly enabled the continuous monitoring of HR [16–18]. The use of such technologies also enables the measurement of HR during sleep [19, 20]. If %HRR with the HR measured during sleep considered as the resting HR is validated, extra efforts taken to accurately measure the resting HR may not be necessary; thus, daily measurement of exercise intensity would be more feasible in the daily clinic. Additionally, this may further enable the 24/7 measurement of exercise intensity.

One of the major effects of exercise is the improvement of fitness, which is linked with the amount of exercise and exercise intensity [21, 22]. To increase the amount of exercise and exercise intensity on a daily basis, not only the scheduled exercise, but also the daily activities other than the scheduled exercise, should be increased. Therefore, continuous monitoring of the activities may be of great significance. If the activities could be measured 24 h a day every day, it would be easier to find a critical and effective solution to increase the amount and intensity of exercise on a daily basis, which will contribute to improve the fitness of the individuals.

In this context, this study aimed to examine the validity of %HRR, calculated with a simplified method using resting HR measured during sleep, against %VO_2_R (the gold standard), in a healthy subject. The measurements were performed during two kinds of exercising; treadmill testing with the Bruce protocol and a 6-min walk test (6MWT). In addition, the feasibility of the 24/7 measurement of exercise intensity with the use of this method was tested in healthy subjects.

## Methods

### Validity study

#### Participants

In the validity study, 12 healthy adults (eight males; mean age, 29 ± 5 years) participated. The individuals 1) without any medical history of diseases which could affect cardiorespiratory fitness and movement function, such as heart failure, myocardial infarction, bone fracture, and spinal cord injury and 2) who agreed to wear the measuring devices during the entire 2-day measurement session after checking the fit of the wearable devices. Non-probability sampling procedures were used to recruit a convenience sample of participants. The exclusion criteria were 1) existence of sleep disturbance and 2) medication which could potentially affect performance. Participants’ height and weight were 166.2 ± 8.6 cm and 59.1 ± 10.8 kg, respectively.

The study protocol was approved by the Medical Ethics Committee of Fujita Health University. All participants provided written informed consent before participation.

#### Procedures and measurements

##### Validity study

In the validity study, each participant performed treadmill testing with the Bruce protocol [4] and a 6MWT. The participants were asked to avoid high intensity exercise and alcohol and caffeine 24 h before the measurement session.

Before treadmill testing and the 6MWT, resting HR and VO_2_ while sitting and while lying were measured after a 10-min sitting interval. Lying and sitting HR were measured with a 5-min interval in a random order to eliminate order effect bias. An average resting HR value of 3 min was used for the analyses. Then, in the 6MWT, participants were instructed to walk at a comfortable speed for 6 min. After a 15-min rest, treadmill exercise testing with the Bruce protocol was conducted. In this experiment, we considered the VO2 and HR to be maximum if the participants satisfied at least three of the following four criteria: 1) maximum voluntary exhaustion as measured by the Borg CR-10 scale; 2) presence of an HR plateau (ΔHR between two consecutive work rates ≤4 beats·min^− 1^); 3) presence of a VO2 plateau (ΔVO2 between two consecutive work rates < 2.1 mL·kg^− 1^·min^− 1^); and 4) a maximal respiratory exchange ratio (RERmax) > 1.1 [10, 23].

Respiratory gas analysis during exercise testing was performed with a Mobile Aero Monitor AE-100i (MINATO Medical Science, Tokyo, Japan). HR was measured using a *hitoe* or ‘smart clothing’ system (NTT corp., Tokyo, Japan and Toray corp. Kyoto, Japan). This consisted of *hitoe* wear, a *hitoe* transmitter, and a smartphone application. An accelerometer embedded in the *hitoe* transmitter estimated trunk posture (lying or not). Participants wore *hitoe* wear during the measurements and the nights before and after the exercise testing. Participants were instructed to go to bed by midnight on these nights. Sleep time was defined as the time when the participant was in the supine position, as judged with the accelerometer, between midnight and 5 a.m. The average of HR between the two nights was used as the sleeping HR. None of the participants reported sleep disorder during the measurement.

#### Analyses

The %HRR was calculated using the equation:

$$ \% HRR=\left( HR- restingHR\right)/\left\{ MaxHR\ during\ treadmill\ testing-\left. restingHR\right\}\right.. $$

In the validity study, HR in a sitting position (HR_sitting_), when lying awake (HR_lying_), or during sleep (HR_sleeping_) were used as the value of resting HR. The actual maximum value (HRmax) was obtained during the treadmill exercise testing.

The %VO_2_R was calculated with the equation:

$$ \%{VO}_2R=\left({VO}_2-{restingVO}_2\right)/\left( Maximum{VO}_2\  during\ treadmill\ testing-{restingVO}_2\right). $$

The VO_2_ value during sitting and maximum VO_2_ value during treadmill exercise testing with the Bruce protocol were used for resting and maximum VO_2_, respectively.

The %HRR and %VO_2_R data used for the analyses were the averaged %HRR and %VO_2_R measured during the middle 1 min of each 3-min stage during the treadmill testing and the averaged %HRR and %VO_2_R measured during the first, third, and the last minutes of the 6MWT. One-way repeated measures analysis of variance (ANOVA) with post-hoc multiple comparisons was performed to examine if there were differences between HR_sitting_, HR_lying_ and HR_sleeping_ values. The agreement of each of these three types of %HRR with %VO_2_R was examined with a Bland-Altman plot [24, 25] for each exercise test.

Fixed and proportional biases were evaluated. Fixed bias was computed as the average difference between %HRR and %VO_2_R, statistically checked by the 95% confidence interval (CI) of the mean differences between the two values ($\overline{\mathrm{d}}$). Fixed bias was indicated if the 95% CI of $\overline{\mathrm{d}}$ did not include zero. Proportional bias was expressed as the correlation coefficient between the difference and average of %HRR and %VO_2_R. When there was proportional bias, the magnitude of the difference between the two values changed depending on the magnitude of the mean of the two values in the Bland-Altman plot. LOA and 95% confidence intervals (CI) around the LOA were calculated using the modified method for a Bland–Altman analysis with multiple observations per individual [26, 27].

Statistical analyses were performed using JMP11 (SAS Institute Inc., Cary, NC, USA). *P*-values < 0.05 were considered statistically significant.

### 24/7%HRR measurement session

#### Participants

In the 24/7 measurement session, three healthy adults (all male, aged 33, 27, and 27, respectively) without any medical history of cardiorespiratory, orthopedic, or neurological diseases participated. The occupations of the participants were medical doctor, physical therapist, and occupational therapist.

#### Procedure

For the 24/7%HRR measurement session, the *hitoe* system was used for continuous monitoring of HR. Each participant wore the *hitoe* wear to monitor HR. For each participant, four pieces of *hitoe* wear were provided, so that they could wash and change the wear. The participants were told that they could take off the wear or transmitter while bathing or whenever they did not want to monitor HR. The observation was performed for consecutive 180 days.

#### Analysis

The %HRR was calculated with HR during sleep as resting HR. The maximum HR was estimated using the Gellish equation (*HRmax* = 206.9 − 0.67 × *age*) [11].

The daily time course of %HRR was compared between working days and holidays. Working days in this experiment were defined as the days when the subjects were at work at for least 8 h a day, while holidays were defined as the days on which the participants were completely off duty. Days with less than 8 h’ work was excluded from the analysis.

The %HRR data was averaged for every 20 s, and then the ensemble average, i.e., the average of each time point through all the observation periods, was calculated for the working days and holidays. The paired t-test was used for comparison between the HR on working days and holidays in each participant.

## Results

### Validity study

The resting HR during sitting, lying, and sleeping were 85 ± 8, 77 ± 9, and 58 ± 5 per minute, respectively.

**Fig. 1:**
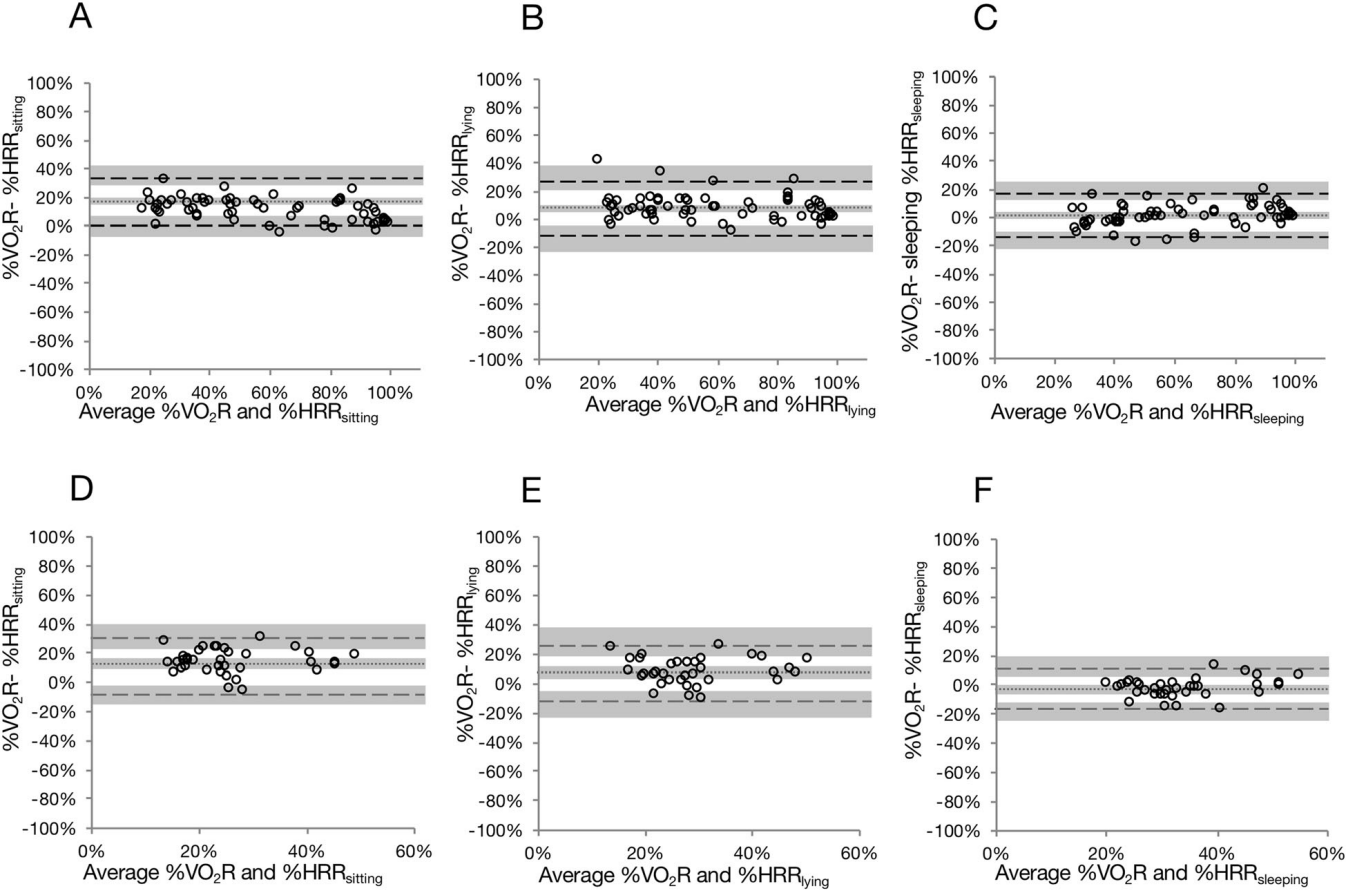
Bland-Altman plots for %VO_2_R and %HRR in treadmill exercise testing and 6-min walk test. **a**, **d**: %VO_2_R vs. %HRR_sitting_; **b**, **e**: %VO_2_R vs. %HRR_lying_; **c**, **f**: %VO_2_R vs. %HRR_sleeping_. Dotted lines represent the average differences between the two methods. Dashed lines represent 95% confidence intervals. Shaded areas represent 95% confidence intervals for mean and limits of agreement. VO_2_R, oxygen consumption reserve; %HRR, percent heart rate reserve; %HRR_sitting_, %HRR calculated with the HR while sitting; %HRR_lying_, %HRR calculated with the HR while lying; awake; %HRR_sleeping_ = %HRR calculated using HR during sleep

The total number of data points used for Bland-Altman plots were 65 in treadmill testing and 36 in 6MWT. HR during sleep (HR_sleeping_) was significantly lower than HR while awake (sitting and lying; HR_sitting_ and HR_lying_). The Bland-Altman plots showed the LOAs between %HRR and %VO_2_R during each exercise test (Fig. 1, Table 1). In the treadmill exercise testing, the mean differences between %VO_2_R and %HRR calculated with HR_sitting_, HR_lying,_ and HR_sleeping_ were 10.8% (95% CI, 8.8 to 12.7%), 7.7% (95% CI, 5.4 to 9.9%), and 1.7% (95% CI, − 0.2 to 3.6%), respectively (Fig. 1a–c). Significant differences between %VO_2_R and %HRR, calculated with HR_sitting_ and HR_lying,_ were observed, indicating fixed bias. There was no significant difference between %VO_2_R and %HRR calculated with HR_sleeping_.

**Table 1:** Fixed and proportional bias in the Bland-Altman analysis

	%VO_2_R vs. %HRR based on	Fixed bias							Proportional bias	
		Mean difference	95% CI	P	Lower	95% CI	Upper	95% CI	difference vs. mean	
					LOA		LOA		r	*P*
Treadmill testing with Bruce protocol	HR_sitting_	10.8	8.8, 12.7	< 0.0001	−5.4	−13.4, 0.0	27.0	22.5, 35.0	−0.45	< 0.01
	HR_lying_	7.7	5.4, 9.9	< 0.0001	−11.4	− 23.5, −4.9	26.8	20.3, 38.8	−0.18	0.14
	HR_sleeping_	1.7	−0.2, 3.6	0.2552	−13.7	−25.3, −9.3	17.2	12.7, 25.3	0.24	0.06
Six minutes walking test	HR_sitting_	12.7	10.0, 15.5	< 0.0001	−5.0	−16.0, 0.0	30.4	24.3, 41.4	0.00	0.98
	HR_lying_	7.0	4.0, 10.0	< 0.0001	−11.6	−23.5, −5.0	25.6	18.9, 37.5	0.02	0.90
	HR_sleeping_	−2.9	−5.0, −0.7	0.0492	−16.3	− 24.6, −11.6	10.5	5.9, 18.9	0.26	0.11

HR, heart rate; LOA, limit of agreement; CI, confidence interval; %VO_2_R, percent oxygen consumption reserve; %HRR, percent heart rate reserve, HR_sitting_; resting HR at sitting awake, HR_sitting_; resting HR at sitting awake, HR_lying_; resting HR at lying awake, HR_sleeping_; resting HR during sleeping

The 95% LOAs between %VO_2_R and %HRRs calculated with HR_sitting_, HR_lying,_ and HR_sleeping_ were − 5.4 to 27.0%, − 11.4 to 26.8%, and − 13.7 to 17.2%, respectively. Proportional bias was found between %VO_2_R and %HRR calculated with HR_sitting_ (r = − 0.45, *P* < 0.01). No significant proportional bias was observed between %VO_2_R and %HRR when calculated with HR_lying_ (r = − 0.18, *P* = 0.14) and HR_sleeping_ (r = 0.24, *P* = 0.06).

In the 6MWT, mean differences between %VO_2_R and %HRR calculated with HR_sitting_, HR_lying,_ and HR_sleeping_ were 12.7% (95% CI, 10.0 to 15.5%), 7.0% (95% CI, 4.0 to 10.0%), and − 2.9% (95% CI, − 5.0% to − 0.7%), respectively. There were significant differences between %VO_2_R and %HRR calculated with HR_sitting_, HR_lying,_ and HR_sleeping_. LOAs between %VO_2_R and %HRR calculated with HR_sitting_, HR_lying,_ and HR_sleeping_ were − 5.0 to 30.4%, − 11.6 to 25.6% and − 16.3 to 10.5%, respectively (Fig. 1d–f). No proportional biases were found between %VO_2_R and %HRR (Table 1).

### 24/7 Measurement session

**Fig. 2:**
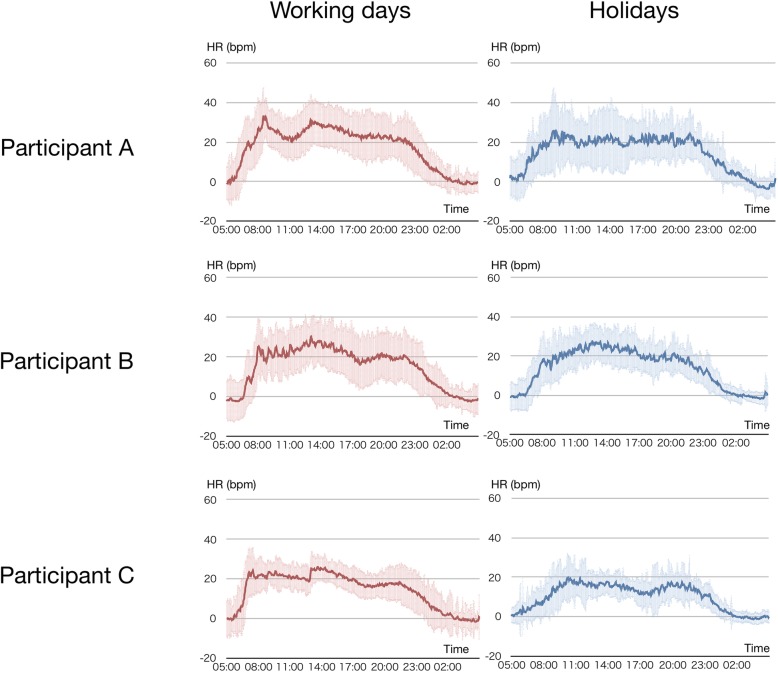
Ensemble average of daily %HRR in working days and holidays of three, participants **a**-**c**. The time course of %HRR of three individuals in working days and holidays are presented. The solid line represents the ensemble average of all the measurement period and the light-colored band represents standard deviation. HR, heart rate; %HRR, percent heart rate reserve

To confirm the feasibility of the 24/7 measurement of exercise intensity with the wearable system and sleeping HR-based exercise intensity measurement, 24/7 measurement of HR in three healthy subjects were performed. The results of 24/7 measurement of HR are shown in Fig. 2. The data was successfully acquired for 132, 142, and 165 days, respectively (working days: 102, 108, 123 days, holidays: 30, 34, 42 days). The graphs show the ensemble average and standard deviation of the %HRR_sleeping_ values of each participant on working days and holidays. In all participants, the average HR values on working days were significantly higher (*P* < 0.0001) than HR values measured on holidays (17.8 ± 14.6 vs 14.6 ± 13.9, 14.6 ± 14.4 vs 12.9 ± 12.6, and 14.5 ± 11.8 vs 10.3 ± 9.7, respectively. The difference in average HR values were more evident during daytime (9 am-5 pm, 26.0 ± 10.9 vs 21.2 ± 12.7 P < 0.0001, 23.7 ± 11.5 vs 21.7 ± 10.2 P < 0.0001, 22.3 ± 6.8 vs 16.3 ± 6.8 P < 0.0001, respectively).

## Discussion

The present results showed that the %HRR with sleeping HR was comparable with % VO_2_R, showing the least amount of errors against the %VO_2_R value among %HRR values investigated in this study. In addition, the experiment monitoring %HRR for 180 consecutive days presents the differences in the daily patterns of exercise intensity between the working days and holidays in each participant, demonstrating the feasibility of this method to monitor exercise intensity.

The results of the current study validated the %HRR_sleeping_ with %VO_2_ R as the gold standard. The results show the possible superiority of %HRR_sleeping_ to %HRR_sitting_, which is more commonly used in exercise intensity calculations. Several factors possibly contribute to these results. First, psychological and physical factors that may influence HR, via the autonomic nervous system [28, 29], are eliminated during sleep. For example, mental stress may result in sympathetic nerve activity and increase HR [30]. Exercise also increases HR, even after cessation of exercise, which is influenced by activity of the sympathetic and parasympathetic nervous systems [31]. The influence of these factors could be removed during sleep. Second, increased stroke volume (SV) in a supine position may contribute to HR better reflecting the change in oxygen consumption. Based on Fick’s principle [32], the relationship between VO_2_ and cardiac output (CO) is described as: VO2 = CO ∗ a − vO2diff (a-vO_2_diff:arteriovenous oxygen difference), where CO is calculated using the equation: *CO* = *SV* ∗ *HR*.

Therefore, the increase in CO during exercise largely reflects the increase in VO_2_. Previous studies have shown that SV increases when starting exercise, but rapidly reaches a plateau [33–35]. Therefore, the increase in CO during exercise is due to an increase in HR and SV. On the other hand, SV also increases during lying rest [33, 34]. If the HR during lying rest is set as baseline, the relationship between HR and CO would less likely be affected by the changes in SV; therefore, HR should be proportional to CO. In combination, these factors possibly affected the superiority of %HRR_sleeping_ observed in the present study. However, the inaccuracy of %HRR_sitting_ in this study was inconsistent with previous studies [10–12], possibly because our results are condition-specific. The present experiment was performed in a hospital environment, and it is possible that the measurements by “white coat” health professionals yielded relatively higher resting HR, as shown in the previous studies [36, 37]. Thus, the measurement errors in HR values taken while patients were awake could be less evident in the other situations. Despite this, the results presented the validity in using resting HR during sleep and indicated that it may be a pragmatic solution for the unpredictable and undesirable variability of resting HR in awake conditions.

As the validity study indicated the validity of the sleeping HR-based exercise intensity calculation, a 24/7 measurement of exercise intensity was performed. The observation of 180 consecutive days of measurement shows the difference in the total amount of exercise intensity between working days and holidays.

The average exercise intensity during daytime ranged from 21 to 26%; this range is consistent with previous studies and demonstrates that the average exercise intensity during working time is approximately 15–30%, which includes different occupations, such as office workers or cleaners [38, 39].

The continuous observation of exercise intensity may enable the accurate estimation of energy expenditure (EE), which would also make the nutrition control more precise. Although previous studies have reported limitations in estimation of EE with wearable devices [40–42], the combination of HR measurement with accelerometry may improve the accuracy of EE estimation with the wearable devices [43, 44].

In addition, the continuous measurement of HR may also be used to manage the amount of exercise performed by rehabilitation patients; for example, continuous HR measurement could be useful in treating patients with problems in exercise tolerance and related functions. Exercise training is considered to improve exercise tolerance function, especially in patients suffering from chronic heart failure, chronic obstructive pulmonary disease (COPD), or chronic kidney failure [45–47]. The combination of using wearable monitoring systems and sleeping HR-based exercise estimation would enable easy monitoring of daily exercise that may possibly improve the management of physical activity and nutrition.

## Limitations

This study has some limitations. First, the *hitoe* wear is relatively tight compared to a usual T-shirt as the electrode should be attached on the skin. This may have affected the HR measurement during sleep. The influence of the comfortability of the wear and HR measurement during sleep should be further investigated.

The sample size of this study may also be considered a limitation. In this study, the number of participants was 12, and for each participant, repeated measurements during treadmill testing and 6MWT were performed. The total number of data points in these experiments with repeated measurement were 64 and 36 in treadmill testing and 6MWT, respectively. The previous studies comparing %HRR and %VO_2_R show strong correlation between these two values; on the other hand, they display a large variety in extent of agreement, which ranges from 20 to 30% at its largest [10, 11]. Considering that the %HRR in this experiment would be at similar levels in terms of agreement with VO_2_R, we set the maximum allowed difference between the methods to 25%. In this study, the pair with smallest difference was the %VO_2_R and %HRR_sleeping_ in treadmill testing, whose mean and standard deviation of the difference were 1.7 and 7.7%, respectively. According to the formula provided by Lu et al., the required sample size for an alpha risk of 0.05 and a power of 80% would be 26, and our sample size satisfies this requirement [48]. There may be still some discussion whether this is really a sufficient sample size, because the sample size is based on the repeated measurements, and not on the individual samples. There is no preceding study on sample size using Bland Altman plot with repeated measurements. Nonetheless, the present result would be at least meaningful to indicate the possible superiority of the sleeping HR-based %HRR calculation to that calculated with sitting HR, which is the widely accepted methodology in clinical practice.

## Conclusions

In the present study, %HRR calculated with the resting HR during sleep was validated with exercise intensity calculations by %VO_2_R. This simplified method may be useful for the daily measurement of exercise intensity using wearable systems, realizing a calibration-less, whole-day measurement model of exercise intensity, which would facilitate further understanding of the effects of exercise on daily activities.

## Data Availability

The datasets used and/or analysed during the current study are available from the corresponding author on reasonable request.

## Abbreviations

%HRR: Percent heart rate reserve
%HRR_lying_: %HRR while lying
%HRR_sitting_: %HRR while sitting
%HRR_sleeping_: %HRR while sleeping
%VO_2_R: Percent VO_2_ reserve
6MWT: 6-min walk test
ANOVA: Analysis of variance
a-VO_2_dif: Arteriovenous oxygen difference
CI: Confidence interval
CO: Cardiac output
COPD: Chronic obstructive pulmonary disease
EE: Energy expenditure
HR: Heart rate
HR_lying_: Heart rate while lying
HR_max_: Maximum heart rate
HR_sitting_: Heart rate while sitting
HR_sleeping_: Heart rate while sleeping
LOA: Limits of agreement
SD: Standard deviation
SV: Stroke volume
VO_2_: Oxygen consumption
